# Activity‐Based Probes for HECT E3 Ubiquitin Ligases

**DOI:** 10.1002/cbic.201700006

**Published:** 2017-06-28

**Authors:** Robert Byrne, Thomas Mund, Julien D. F. Licchesi

**Affiliations:** ^1^ Department of Biology and Biochemistry University of Bath Claverton Down Bath BA2 7AY UK; ^2^ MRC Laboratory of Molecular Biology Francis Crick Avenue Cambridge Biomedical Campus Cambridge CB2 0QH UK

**Keywords:** activity-based probes, biological activity, HECT E3 ubiquitin ligase, proteasome, protein–protein interactions, ubiquitin

## Abstract

Activity‐based probes (ABPs) have been used to dissect the biochemical/structural properties and cellular functions of deubiquitinases. However, their utility in studying cysteine‐based E3 ubiquitin ligases has been limited. In this study, we evaluate the use of ubiquitin‐ABPs (Ub‐VME and Ub‐PA) and a novel set of E2–Ub‐ABPs on a panel of HECT E3 ubiquitin ligases. Our in vitro data show that ubiquitin‐ABPs can label HECT domains. We also provide the first evidence that, in addition to the RBR E3 ubiquitin ligase Parkin, E2–Ub‐ABPs can also label the catalytic HECT domains of NEDD4, UBE3C, and HECTD1. Importantly, the endogenous proteasomal E3 ligase UBE3C was also successfully labelled by Ub‐PA and His‐UBE2D2–Ub‐ABP in lysate of cells grown under basal conditions. Our findings provide novel insights into the use of ABPs for the study of HECT E3 ubiquitin ligases.

## Introduction

Ubiquitin (Ub) is a small (76 amino acids) and highly conserved protein, best known for its role in mediating protein degradation as part of the ubiquitin–proteasome system.^1^, ^2^ It is conjugated through an enzymatic cascade involving an E1‐activating enzyme (E1), E2‐conjugating enzymes (E2s), and E3 ubiquitin ligases (E3 ligases) onto lysine(s) of a protein target.^3^, ^4^ During the ubiquitin cascade, the C‐terminal Gly76 residue of Ub is adenylated by an E1 in an ATP‐dependent reaction prior to the molecule being transferred onto the catalytic cysteine (Cys) residue of the E1. Ub is then transferred by a *trans*‐thioesterification reaction onto the catalytic Cys residue of an E2. E3 then facilitates the transfer of ubiquitin from E2 onto the substrate. E3 ligases are categorised into different groups based on the mechanism by which they catalyse this transfer: RING,^5^ U‐box,^6^ ring‐between‐ring (RBR),^7^ and HECT.^8^ HECT and RBR accept Ub on a catalytic Cys residue, whereas RING and U‐box ubiquitin ligases act as scaffolds to provide optimal orientation for the transfer of ubiquitin from E2 to the substrate. The RBR E3s represents an unusual hybrid family, and shares features with RING and HECT ligases. In RBR ligases, the enzyme activity is contributed to by an intrinsic C‐terminal domain, but is also able to recruit thioesther‐bound E2 enzymes at a RING domain.^7^

**Figure 1:**
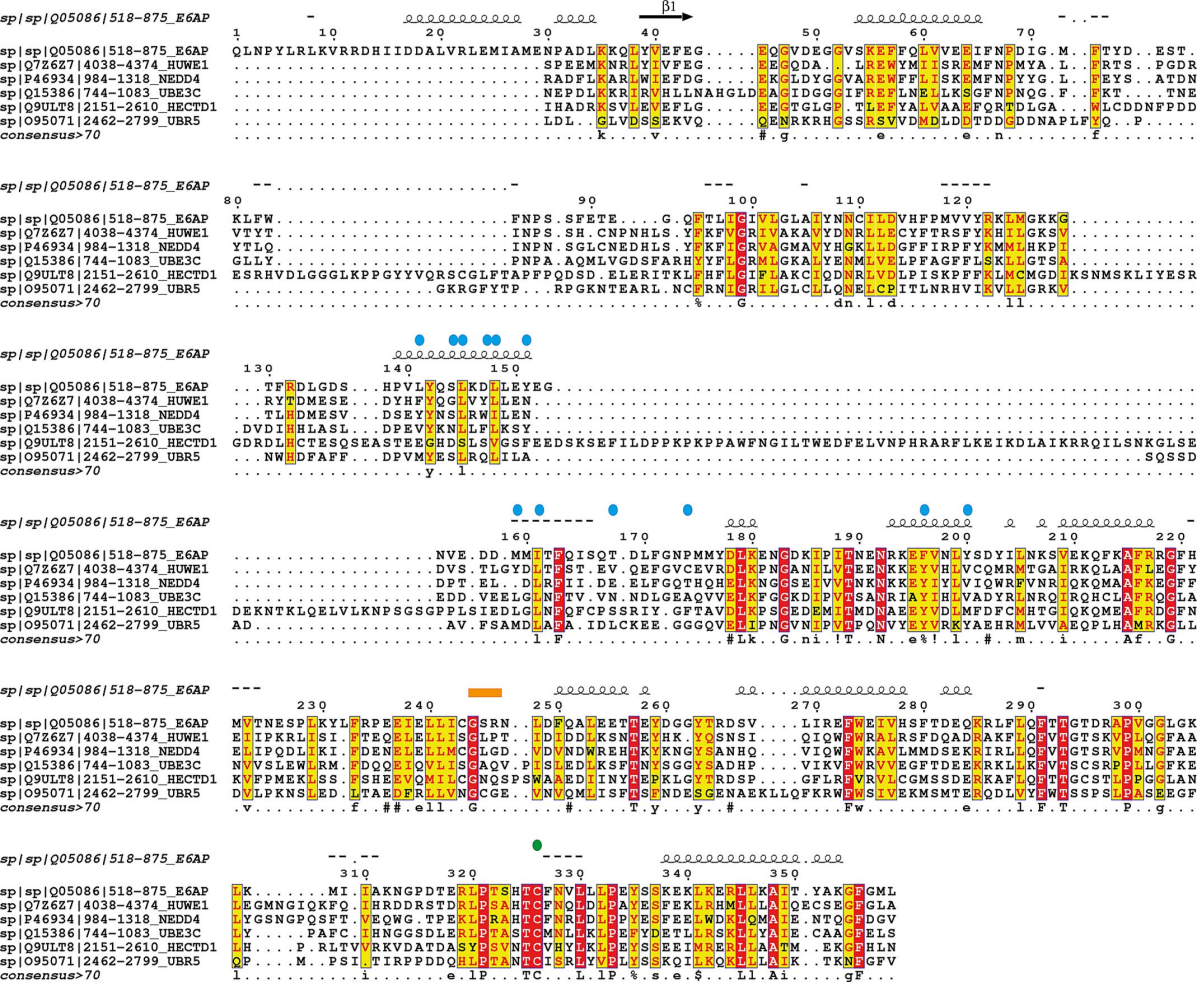
Sequence alignment of HECT domains of human E6AP, HUWE1, NEDD4, UBE3C, HECTD1, and UBR5. Residues strictly conserved (red boxes) and partially conserved (yellow boxes) are shown. Blue circles indicate residues mediating E2 binding in the structure of E6AP:UBCH7;^10^ the conserved catalytic cysteine is shown as a green circle, and the flexible hinge loop connecting the N and C lobes is shown as an orange box.

The HECT family was first discovered when investigating the function of E6AP, a protein that forms a complex with human papillomavirus E6 oncoprotein types 16 and 18.^9^ HECT E3 ligases feature a highly conserved C‐terminal HECT domain of around 350 amino acids; this consists of the E2‐binding N lobe separated from a C lobe (containing the active‐site Cys) by a short hinge loop that provides flexibility (Figure 1).^10^ The conserved catalytic Cys receives Ub from the E2 prior to establishing an isopeptide bond between the C‐terminal Gly76 of Ub and a Lys ϵ‐amino group on the target protein.^11^ The HECT family contains 28 members in humans and, based on the N‐terminal domain architecture, can be subdivided into three groups:^12^ NEDD4 (neural precursor cell‐expressed developmentally downregulated 4) has nine members; the HERC subfamily has six members; the other subfamily is composed of 13 members including the candidate ubiquitin‐fusion degradation E3 ubiquitin ligases TRIP12,^13^ HECTD1,^14^ and HUWE1 (Mule),^15^ the proteasomal E3 ligase UBE3C (KIAA10),^16^ and the N‐end‐rule pathway E3 ligases, UBR1‐7.^17^ Recently, phylogenetic analysis led to the classification of HECT E3 ubiquitin ligases into class I to class VI.^18^, ^19^ Some HECT ligases are involved in the DNA damage response, apoptosis, and cell proliferation; this is interesting given that HUWE1, UBR5, and NEDD4 are overexpressed in certain cancers.^20^ Therefore, targeting the Cys‐based catalytic activity of enzymes in the ubiquitin system represents a new and exciting avenue for anticancer therapy.

To this end, the expansion of the Ub toolbox with activity‐based probes (ABPs) has contributed to a rapid increase in our understanding of deubiquitinases (DUBs).^21^, ^22^ First‐generation ABPs (Ub‐ABPs) comprise a Ub for selective recognition by DUBs, with the C‐terminal G76 chemically modified with an electrophilic warhead to covalently label the catalytic Cys. Ub‐ABPs have been designed with different C‐terminal warheads (aldehyde,^23^ vinyl sulfone,^24^ vinyl methylester (Ub‐VME),^25^ and propargylamide (Ub‐Prg or Ub‐PA)).^26^ These have been used to identify novel DUB families^25^ and to monitor DUB activity.^27^, ^28^, ^29^ In addition, they can be used as powerful structural tools for profiling DUB inhibitors and to dissect the mechanisms of action of DUBs.^30^, ^31^ For example, the identification of S1 and secondary S2 ubiquitin‐binding sites on the catalytic domain of USP21^32^ and OTU (ovarian tumour) family DUBs^33^, ^34^ has led to a new generation of Ub‐ABPs based on diubiquitin.^35^

In contrast to DUBs, the Ub toolbox remains largely underexplored for Cys‐based E3 ubiquitin ligases. E3 ubiquitin ligases have been proposed to contain less reactive Cys nucleophiles, thus suggesting that these enzymes might be less reactive to existing Ub‐ABPs.^26^ However, recent evidence suggests that C‐terminal electrophilic probes can indeed label Cys residues in HECT ligase. Structural studies have shown that the HECT domain can be bound by Ub on its C lobe. Furthermore, the flexibility between the C and N lobes, important for the catalytic function of HECT domains, can orientate the C‐terminal electrophilic warhead of Ub‐ABPs and lead to labelling of the catalytic Cys residue in HECTs, in addition to DUBs.^36^, ^37^, ^38^ For example, the recombinant HECT domain of HUWE1 was found to react with the Ub‐VME‐ABP, and interestingly multiple Cys residues on the HECT domain were found to be labelled by the probe.^39^ Importantly, mutation of probe‐labelled Cys residues resulted in reduced, but not total loss of, ubiquitin ligase activity, thus suggesting that Cys residues other than the catalytic Cys might contribute to enzymatic activity. Furthermore, HUWE1, E6AP, and TRIP12 were labelled by Ub‐VME by using mouse and human cell‐line lysates.^39^ More recently, recombinant HUWE1 was labelled by Ub‐PA.^26^ However, as E2–Ub is the native substrate of E3s, Ub‐ABPs do not necessarily provide a mechanistically relevant measure of E3 activity; we question the biological validity of using these probes for studying E3 ligases. The recent development of E2‐based ABPs (referred to here as E2–Ub‐ABPs or E2–ABPs) that mimic a ubiquitin‐charged E2 with a C terminal electrophilic warhead has provided further selectivity for the labelling of catalytic Cys, in particular for RBR and HECT E3 ubiquitin ligases.^40^ E2–Ub‐ABPs can be customised with defined E2 components.^41^ The activated vinylsulfide warhead employed is also less electrophilic. These properties enabled E2–Ub‐ABPs to be successfully used to profile the transthiolation of the RBR E3 ligase Parkin, NEDD4L, and a bacterial HECT‐like E3 ligase.^40^ The crystal structure of the HECT domain of NEDD4L in complex with E2‐ubiquitin shows how the C terminus of Ub is positioned between UBE2D2 (bound to the N lobe) and the active‐site Cys in the C lobe.^37^, ^38^ This therefore, makes E2–Ub‐ABPs more specific in targeting the catalytic Cys residues of HECT ligases compared to Ub‐ABPs.

In order to address the shortcomings of existing Ub‐ABPs for studying E3 ligases, a probe with a C‐terminal dehydroalanine (Dha) residue was developed.^42^ Dha retains a native carboxy terminus thus allowing it to be processed by the native Ub conjugation machinery with E1–Ub‐based and E2–Ub‐based probes transiently formed in situ. This allows covalent labelling of all components of the Ub conjugation machinery. Activity‐based proteomics using Ub‐Dha confirmed that this probe can indeed label E1 enzymes, E2s, some OTU DUBs (e.g., OTUB1, Atnx3, Usp14, and Uchl1) and a range of E3s including NEDD4 family members (e.g., NEDD4, Smurf2, WWP1 and 2), HECTD1, UBE3A, UBE3C, and TRIP12.^42^ However, compatibility with the Cys‐rich RBR group of E3 ligases was not demonstrated.

In this study, we evaluated the use of Ub‐ABPs (Ub‐VME and Ub‐PA) and E2–Ub‐ABPs on a panel of HECT ligases (NEDD4, UBE3C, HUWE1, HECTD1, and UBR5). Our work demonstrates that both types of ABPs can be used effectively to study HECT E3 ligases, either when expressed recombinantly in vitro or at endogenous levels in cell lysates in the case of UBE3C.

## Results

We aimed to determine which ABPs could be used for HECT E3 ubiquitin ligases: HECTD1 (class II), UBR5 (class IV), UBE3C (class V), NEDD4 (class VI), and HUWE1 (class VI).^19^ These HECT ligases show high conservation (Figure 1) of the N‐ and C lobes, the hinge loop connecting the N‐ and C lobes, as well as the catalytic Cys. We first used in vitro auto‐ubiquitylation to assess whether GST‐tagged HECT ligases domains (GST‐HECT) expressed in *Escherichia coli* were active. In the absence of substrates, HECT ligases can auto‐ubiquitylate with Ub. We therefore used this to determine whether the recombinant GST‐HECTs were functional and active. E2‐binding elements originally mapped on E6AP differ between HECT ligases, but they retain the hydrophobic nature, which is key for E2 binding.^10^ We included HUWE1 because it is the only HECT E3 ubiquitin ligase shown to be labelled by ubiquitin‐VME^39^ and ubiquitin‐PA.^26^

**Figure 2:**
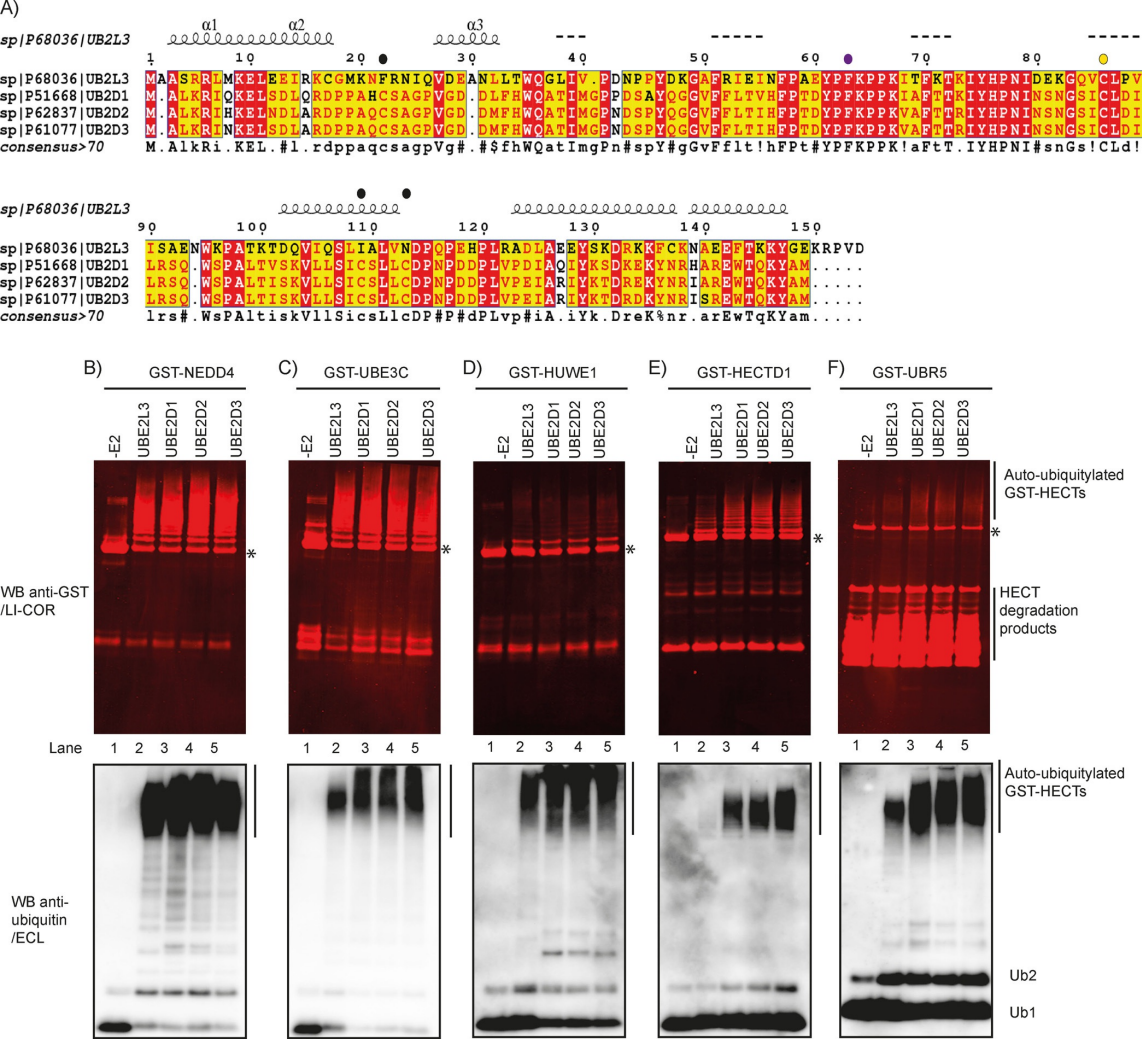
Compatibility of E2s to support HECT ligase activity. A) Sequence alignment of UBE2L3 (UBCH7) and UBE2D family members UBE2D1 (UBCH5A), UBE2D2 (UBCH5B), and UBE2D3 (UBCH5C). Residue F63 (UBE2L3), critical for binding to the HECT domain of E6AP and conserved between UBE2L3 and UBE2Ds (F62), is shown as a purple circle. Noncatalytic Cys residues are shown as black circles; catalytic Cys is shown as a yellow circle. Auto‐ubiquitylation assays were carried out with WT ubiquitin, His‐E1, E2, and tagged HECT catalytic domains: B) GST‐NEDD4, C) GST‐UBE3C, D) GST‐HUWE1, E) GST‐HECTD1, and F) GST‐UBR5. After 3 h at 30 °C, reactions were terminated by addition of 4×LDS/DTT and resolved on 4–12 % SDS PAGE gels. Auto‐ubiquitylation was detected with an anti‐GST antibody followed by LI‐COR infrared detection (top panels) or with an anti‐ubiquitin antibody followed by ECL detection (lower panels). Reactions lacking E2 (‐E2) were negative controls. Asterisks indicate unmodified GST‐HECT domains.

First, we explored whether UBE2L3 and UBE2D2 showed any preference for supporting the ligase activity of specific HECTs. UBE2L3 (UBCH7) and the UBE2D family E2s show conservation of the catalytic Cys (C86 in UBE2L3, C85 in UBE2Ds), as well as conservation of the critical residue for binding to the N lobe of HECT ligases (F63 in UBE2L3 and F62 in UBE2Ds; Figure 2 A).^43^ UBE2L3 has been shown to support the activity of HECT ligases including NEDD4, E6AP, and UBE3C.^44^, ^45^, ^46^ In agreement with published work, UBE2L3 supported the E3 ubiquitin ligase activity of GST‐NEDD4, GST‐UBE3C, and GST‐HUWE1 (Figure 2 B–D). Furthermore, UBE2L3 supported the activity of GST‐UBR5 (Figure 2 F). Although UBE2L3 led to auto‐ubiquitylation of GST‐HECTD1 (Figure 2 E, lane 2, upper panel), it showed weak functional interaction, as shown by the absence of an auto‐ubiquitylated smear (Figure 2 E, lane 2, lower panel). This interesting observation emphasises the need to understand better E2:HECT pairs.^47^ In contrast to UBE2L3, all UBE2D family members (UBE2D1–3) were able to support the activity for all GST‐HECTs in our panel. Although GST‐HUWE1 activity was evident as a strong ubiquitylated smear, there was only a main higher molecular weight band and a weak smear by detection with GST. This could be due to the fact that the GST tag might be modified with ubiquitin thereby affecting recognition of the epitope by the anti‐GST antibody.

**Figure 3:**
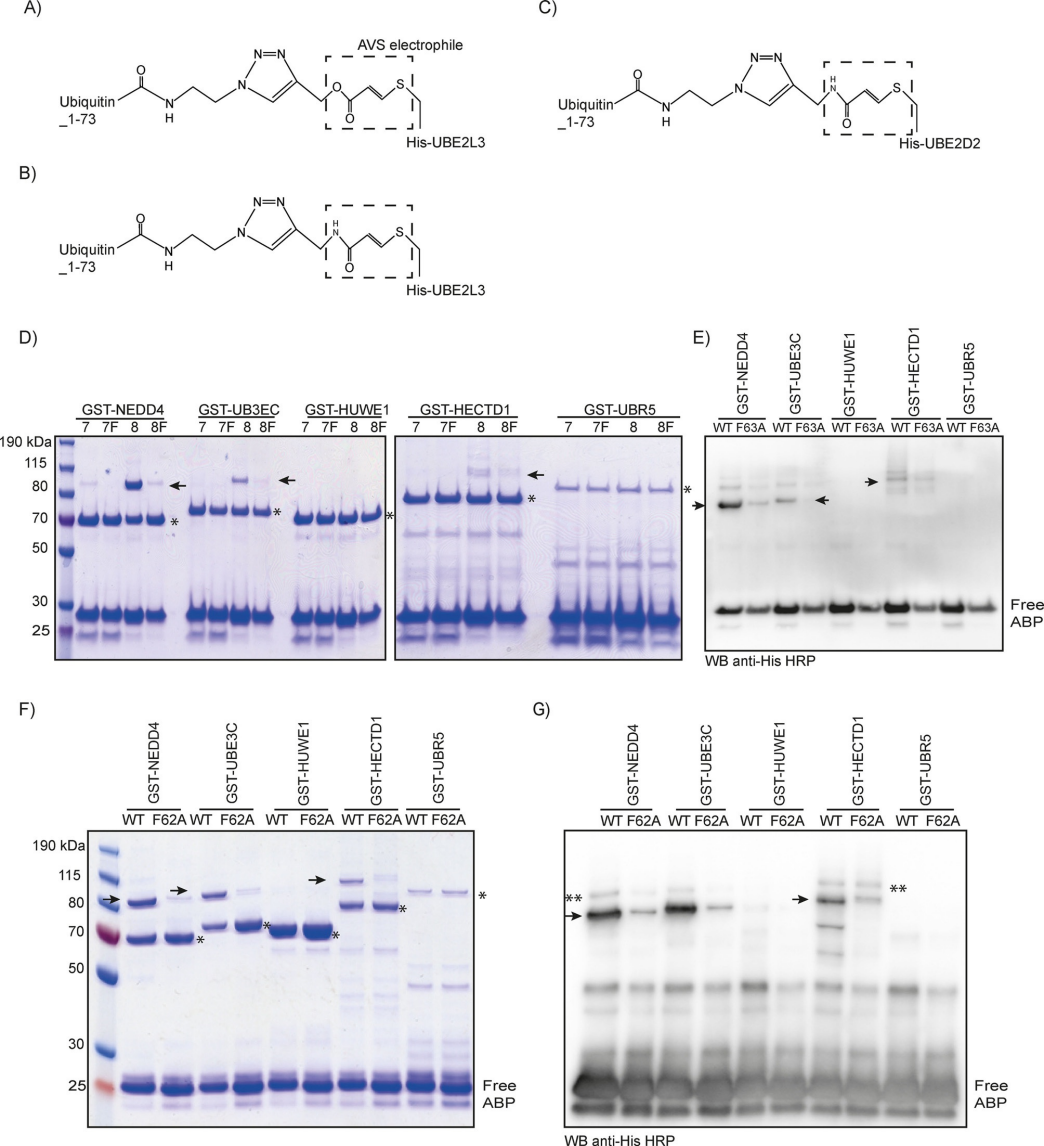
In vitro labelling of HECT domains with His‐UBE2L3–Ub and His‐UBE2D2–Ub‐ABPs. A), B) Chemical structure of first‐generation E2–ubiquitin‐ABPs engineered with His‐UBE2L3 (His‐UBE2L3–Ub‐ABP, referred to as His‐UBE2L3‐ABP). Two such probes, A) **7** and B) **8**, differ in their warhead; **8** is more reactive.^40^ C) His‐UBE2D2–Ub‐ABP (His‐UBE2D2‐ABP) has a similar warhead to **8** but more closely mimics the native ubiquitin C terminus. D) GST‐HECT domains were incubated for 8 h at 30 °C with either WT (**7** and **8**) or F63A mutants (**7 F** and **8 F**). Mutant probes containing His‐UBE2L3_F63A (**7 F** and **8 F**) and His‐UBE2D2_F62A are binding‐deficient mutants that were used to show specificity for the probes in labelling the E3 ligases.^40^ Reactions were terminated by addition of 4×LDS/DTT and analysed on a 4–12 % SDS PAGE gel stained with Coomassie. Single asterisks indicate unlabelled GST‐HECT domains; black arrows indicate ABP‐labelled GST‐HECT domains. E) These reactions were also analysed by western blotting with an anti‐His HRP antibody to detect free probe and probelabelled GST‐HECTs (arrows). GST‐HECT domains were incubated for 8 h at 30 °C with His‐UBE2D2_WT‐ABP or His‐UBE2D2_F62A‐ABP and analysed by F) Coomassie stain or G) western blot with HRP‐conjugated anti‐His antibody. Double asterisks indicate background bands representing not fully reduced samples or minor secondary Cys labelling sites.

Having confirmed that recombinantly expressed GST‐HECTs were active, we screened a different set of ABPs for their potential use in labelling the catalytic domain of five HECT ligases. We first tested E2–Ub‐ABPs (Figure 3).^40^ These novel probes have been successfully used to label the catalytic Cys of the RBR E3 ubiquitin ligase Parkin.^40^ We tested the ability of two E2–Ub‐ABPs probes (**7** and **8**) for labelling recombinant GST‐HECTs. Probes **7** (Figure 3 A) and **8** (Figure 3 B) are two variants of a probe design. They differ in the electrophilic warhead, with probe **8** being more reactive. This might be explained by increased electrophilicity, or that its C terminus more closely mimics the native Ub C terminus, or both. As expected, incubation of GST‐HECTs with these probes revealed that GST‐NEDD4 and GST‐UBE3C showed a strong labelling with **8** and weaker labelling with **7**. Importantly, the labelling was markedly decreased with the corresponding F63A mutant probes (**7 F** and **8 F**) for both GST‐NEDD4 and GST‐UBE3C, thus validating the specificity of each His‐UBE2DL3–Ub‐ABP. In contrast, no labelling was observed for either GST‐HUWE1 or GST‐UBR5, whereas a weak labelling signal was obtained with GST‐HECTD1 (Figure 3 D). Data obtained with Coomassie staining was confirmed by immunoblotting with an HRP‐conjugated anti‐His antibody that specifically detected free ABPs as well as ABP‐labelled GST‐HECT domains of NEDD4 and UBE3C, and to a lesser extent GST‐HECTD1 (Figure 3 E).

Given that UBE2L3 only weakly supports GST‐HECTD1 auto‐ubiquitylation (Figure 2 E), we next tested whether an E2–Ub‐ABPs based on UBE2D2 (Figure 3 C), which efficiently supports HECTD1 ligase activity (Figure 2 E, lane 4), would label GST‐HECTD1 more efficiently. Recombinant GST‐HECT domains were incubated with either His‐UBE2D2_WT‐ABP or His‐UBE2D2_F62A‐ABP. Similarly to the His‐UBE2L3_WT‐ABP (**8**), GST‐NEDD4 and GST‐UBE3C were efficiently labelled when using His‐UBE2D2_WT_ABP but not with the corresponding HECT‐binding‐deficient mutant probe (His‐UBE2D2_F62A‐ABP). No labelling could be detected for GST‐HUWE1 or GST‐UBR5, whereas GST‐HECTD1 showed strong and specific labelling with His‐UBE2D2_WT‐ABP (Figure 3 F). Labelling reactions were also detected with an anti‐His antibody, which showed clear specific labelling for GST‐NEDD4, GST‐UBE3C, and GST‐HECTD1, with the strongest band corresponding to the expected shift in molecular weight following probe labelling (Figure 3 G).

**Figure 4:**
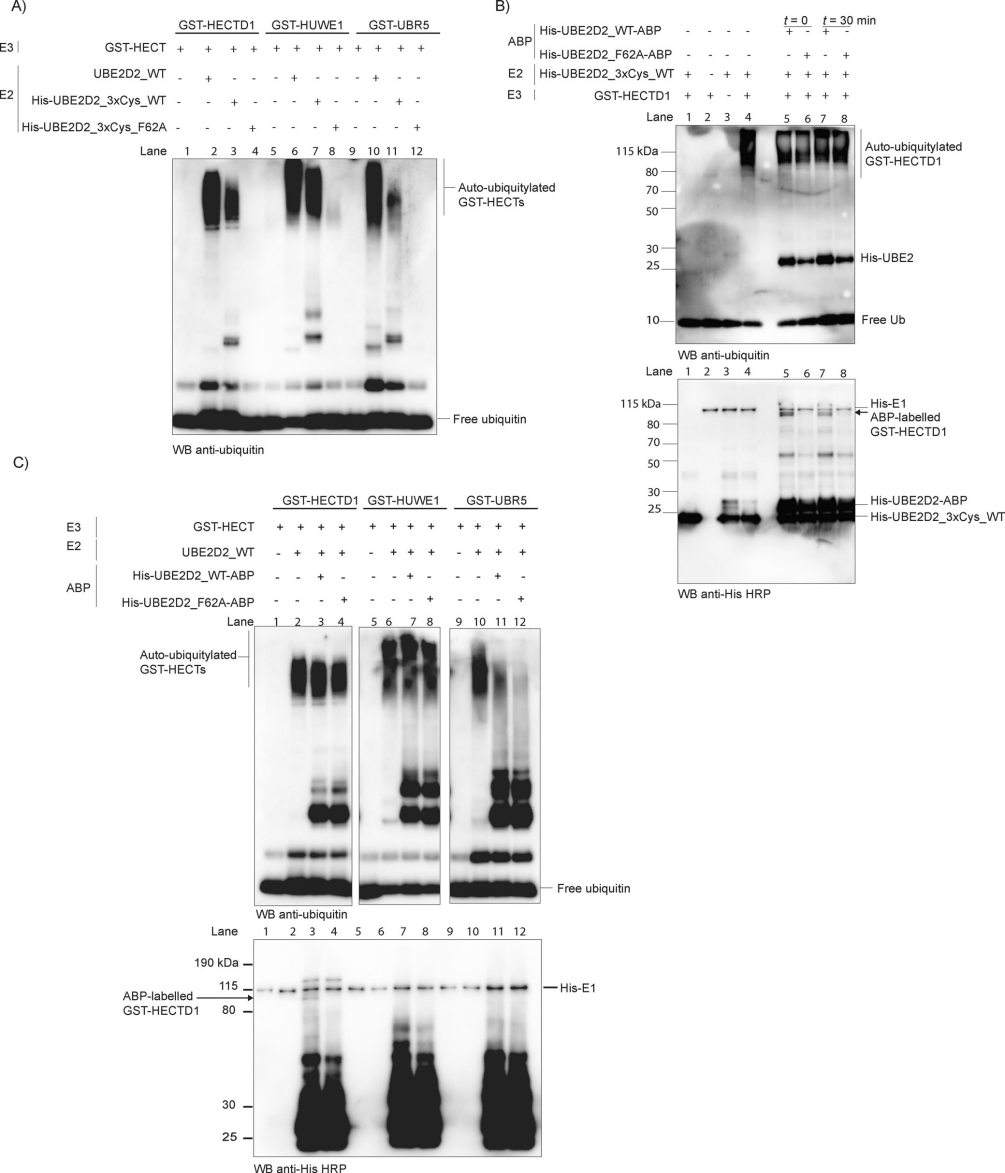
Labelling of GST‐HECTD1 by auto‐ubiquitylation assay. A) Auto‐ubiquitylation assays with GST‐HECT domains and either no E2 (lane 1), wild‐type UBE2D2 (lane 2), His‐UBE2D2_3xCys_WT (noncatalytic Cys mutated: C21S, C107S and C111S) or His‐UBE2D2_3xCys_F62A (C21S, C107S, and C111S; F62A: binding mutant). Auto‐ubiquitylation was detected with a polyclonal rabbit anti‐ubiquitin antibody. B) Auto‐ubiquitylation assays for His‐E1, His‐UBE2D2_3xCys_WT, GST‐HECTD1, Ub, and either His‐UBE2D2_WT‐ABP or His‐UBE2D2_F62A‐ABP. ABPs were added either immediately or after 30 min. Products were detected with a polyclonal anti‐ubiquitin antibody (above) and an anti‐His HRP antibody (below). Lanes 1–3 show control reactions (No E1, No E2, and No E3, respectively). The smear in lane 4 represents HECTD1 ubiquitin ligase activity. Lanes 5–8 show that addition of His‐UBE2D2_WT‐ABP (but not mutant F62A) specifically labels HECTD1, based on molecular weight estimation (arrow, lanes 5 vs. 6, 7 vs. 8). The expected molecular weight of His‐E1 is 118 kDa; GST‐HECTD1 labelled with His‐UBE2D2_WT‐ABP is 106 kDa. Note that anti‐His HRP also detects His‐tagged E1 and E2 as indicated. C) As in B) but with UBE2D2_WT as the E2 (immediate addition of ABP). In contrast to GST‐HECTD1, no labelling was observed (neither GST‐HUWE1 nor GST‐UBR5).

In order to address the lack of labelling of GST‐HUWE1 and GST‐UBR5 with E2–Ub‐ABPs, we next tested whether probe labelling is more efficient under conditions of active ligase activity. In order to ensure that the modified E2 used for E2–Ub‐ABPs still supports HECT ligase activity, we first carried out in vitro auto‐ubiquitylation with UBE2D2, or modified versions of this E2 in which the three noncatalytic Cys residues are mutated (C21S, C107S, and C111S): His‐UBE2D2_3xCys_WT and His‐UBE2D2_3xCys_F62A (Figure 4 A). These assays revealed that His‐UBE2D2_3xCys_WT supports HECTD1 and HUWE1 but not UBR5 ligase activity (Figure 4 A, lane 11). In agreement with our previous data, His‐UBE2D2_3xCys_F62A did not support HECT ligase activity (Figure 4 A, lanes 4, 8, and 12). We next tested whether addition of the probe at the start of the auto‐ubiquitylation assay or 30 min later affected the labelling of HECTD1. Although we detected clear labelling of GST‐HECTD1 with His‐UBE2D2_WT‐ABP (but not with the F62A mutant), the time at which the probe was added had no effect on labelling (Figure 4 B, bottom panel, lane 7 vs. 5, arrow). Interestingly, although ABPs act as suicide probes of DUBs, we did not detect any decrease in the activity of HECTD1 (Figure 4 B, top panel, lane 6 vs. 5; lane 8 vs. 7). This is likely due to the fact that only a small proportion of the enzyme is rendered inactive as a result of labelling. We expanded this assay to GST‐HUWE1 and GST‐UBR5 but found no evidence of labelling for either enzyme, although we still detected labelling for GST‐HECTD1 (Figure 4 C bottom panel, lane 3, arrow). The decrease in UBR5 activity in the presence of His‐UBE2D2_WT‐ABP is intriguing (Figure 4 C, top panel, lane 11 vs. 10). However given that the wild‐type and mutant ABPs show similar patterns (Figure 4 C, top panel, lane 11 vs. 12), this strongly suggests that the effect is independent of the functionality of the probe.

**Figure 5:**
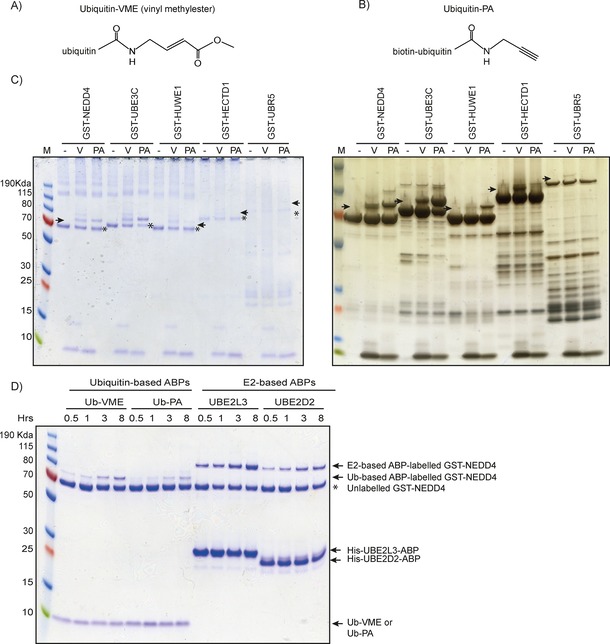
In vitro labelling of GST‐HECT domains with Ub‐based ABPs, A) Ub‐VME,^25^ or B)  Biotin‐Ub‐PA (also referred to as Ub‐PA).^26^ C) GST‐tagged NEDD4, UBE3C, HUWE1, HECTD1, or UBR5 was incubated for 8 h at 30 °C with either Ub‐VME (V) or Ub‐PA (PA). Reactions were resolved on a 4–12 % PAGE and detected by Coomassie (left) or silver staining (right). Asterisks indicate unlabelled GST‐HECT; arrows indicate GST‐HECT domains labelled by Ub‐ABP. D) Time‐course [hours] of labelling of GST‐NEDD4 at 30 °C with Ub‐ABPs and E2–Ub‐ABP.

Given previous reports that Ub‐VME and Ub‐PA can label HUWE1, we tested these ABPs on NEDD4, HECTD1, UBR5, and UBE3C. The main functional difference between these two probes is the C‐terminal electrophilic warhead (Figure 5 A and B), which is more electrophilic in the case of Ub‐PA.^26^ Recombinant GST‐HECT domains were incubated with 10 μm Ub‐VME or Ub‐PA and analysed by SDS‐PAGE gel stained by Coomassie or silver stain (Figure 5 C). As previously shown by others, GST‐HUWE1 was successfully labelled with Ub‐PA.^26^ We also observed labelling of GST‐NEDD4 and GST‐UBE3C but not of GST‐UBR5 or GST‐HECTD1.

Also in agreement with previously results, we observed some (albeit weak) labelling of GST‐HUWE1 with Ub‐VME,^39^ as well as labelling of GST‐NEDD4, GST‐UBE3C, GST‐HECTD1, and GST‐UBR5 (Figure 5 C). In order to directly compare these probes, we carried out a time‐course experiment (Figure 5 D); this revealed quick and efficient labelling of GST‐NEDD4 by E2‐based ABPs.

**Figure 6:**
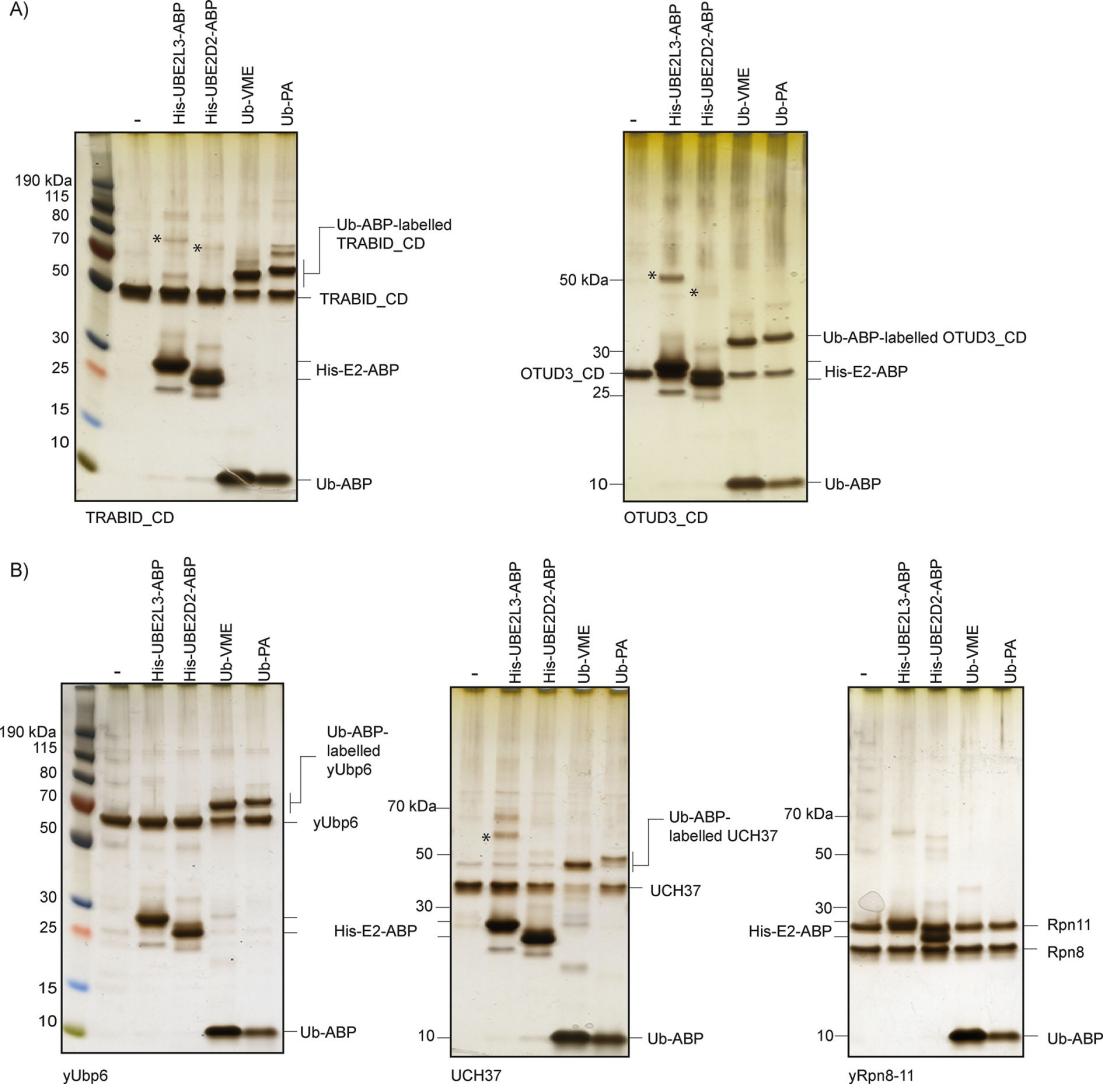
His‐E2–Ub‐ABPs do not label proteasomal DUBs. In vitro labelling of A) TRABID catalytic domain (CD; AnkOTU) and OTUD3‐CD, and B) proteasomal DUBs yUbp6, UCH37, and yRpn8‐11 with the indicated ABPs. DUBs were incubated for 8 h at 30 °C with His‐UBE2L3‐ABP, His‐UBE2D2‐ABP, Ub‐VME, or Ub‐PA. Products were resolved on a 4–12 % PAGE and detected by silver staining. Asterisks indicate weak background labelling of DUBs with His‐E2–Ub‐ABP, based on molecular weight shift.

In order to determine the specificity of ABPs, we analysed the labelling of DUBs, including the catalytic domain (CD) of TRABID (AnkOTU)^48^ and of OTUD3. We observed strong labelling of TRABID_CD and OTUD3_CD with Ub‐VME and Ub‐PA as expected, and virtually no labelling with His‐UBE2L3‐ or His‐UBE2D2‐ABPs (Figure 6 A). In order to provide a specific context where the labelling of HECTs over DUBs might be important, we next determined the specificity of ABPs on three proteasomal DUBs: yUbp6, UCH37, and yRpn8‐11.^49^ We found that yUbp6 and UCH37 could be labelled by Ub‐ABPs but not by E2–Ub‐ABPs. Specifically, yUbp6 was efficiently labelled with Ub‐VME and Ub‐PA, whereas UCH37 was preferentially modified by Ub‐VME. The heterodimer yRpn8‐11, which contains the Zn^2+^‐dependent DUB Rpn11, was used as a negative control as its DUB activity is not Cys‐based.^50^ Importantly, neither His‐UBE2D2‐ABP nor His‐UBE3L3‐ABP convincingly labelled proteasomal DUBs (Figure 6 B). This, together with the fact that E2–Ub‐ABPs efficiently modified the HECT domain of the proteasomal E3 ubiquitin ligase UBE3C (Figure 3), suggests that the specificity of E2–Ub‐ABPs could be particularly useful for dissecting the activity and function of Cys‐based E3 ubiquitin ligases, for example, in the context of the proteasome, where both E3 ligases and DUBs are present.^51^

**Figure 7:**
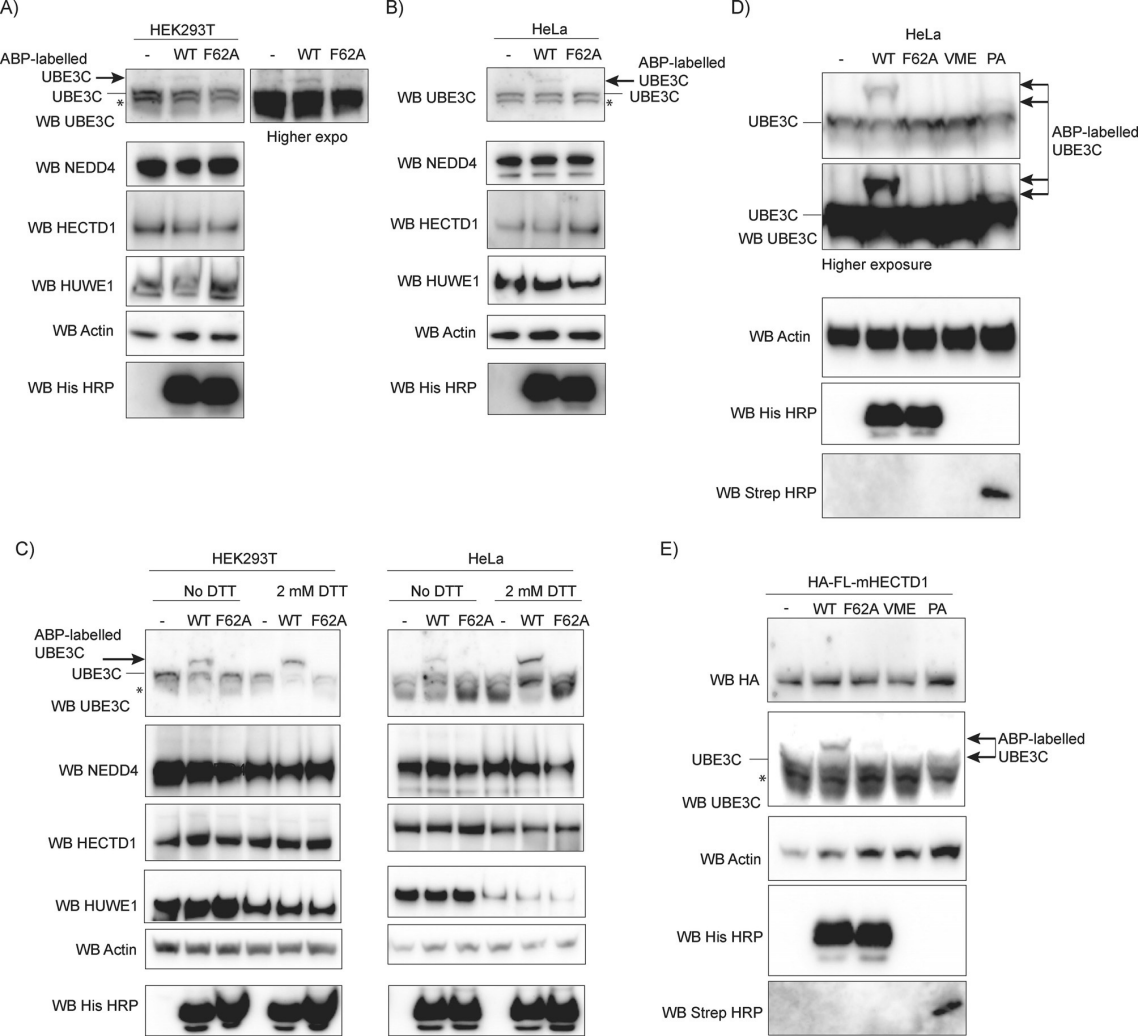
ABP labelling of endogenous UBE3C in cell lysate. Lysates from HEK293T and HeLa cells were incubated for 3 h with 5 μm probe and analysed by western blotting (WB specifies antibody). A shift in the molecular weight of the ligase indicates labelling (arrows). An asterisk indicates a contaminating band detected by the antibody. Weak yet specific labelling of endogenous UBE3C with His‐UBE2D2_WT‐ABP (but not the F62A mutant) was detected under basal/unstimulated conditions in A) HEK293T and B) HeLa cells lysed in buffer with a 0.5 % Triton X‐100. An HRP‐conjugated His antibody was used as a loading control for His‐UBE2D2‐ABPs. In contrast to UBE3C, labelling of other HECT ligases was not detected. C) Western blot shows improved labelling of endogenous UBE3C under basal/unstimulated condition after sonication (no detergent). Addition of 2 mm DTT during labelling resulted in a slight increase in labelling of endogenous UBE3C. Similarly to A) and B), no labelling is detected for other HECT ligases. D) Western blot analysis of HeLa cell lysates obtained by sonication and incubation with E2–Ub‐ABPs (His‐UBE2D2_WT‐ABP and the F62A mutant) or Ub‐ABPs (Ub‐VME and Ub‐PA). As in C), UBE3C was efficiently labelled by His‐UBE2D2_WT‐ABP. We also observed some weak labelling with biotin‐Ub‐PA but not with Ub‐VME. HRP‐conjugated His and streptavidin‐HRP antibodies were used as loading control for the probes. Note that Ub‐VME did not have a tag. E) Cell lysate from HEK293T overexpressing HA‐tagged full‐length‐mouse HECTD1 was incubated with ABPs. UBE3C was used as a positive control for probe functionality. However, labelling of HA‐FL‐mHECTD1 was not detected in this assay.

Having established that E2–Ub‐ABPs and Ub‐ABPs can label the HECT domains of a panel of E3 ubiquitin ligases, we next set out to determine the use of these probes in a cellular context. Previous studies have reported that E6AP and TRIP12 can be labelled by Ub‐VME,^39^ whereas HUWE1 was shown to be labelled by both Ub‐VME^39^ and Ub‐PA.^26^ Although the recombinant HECT domain of NEDD4L, the RBR domain of HOIP, and the bacterial HECT‐like E3 ligase NleL were shown to be labelled by E2–Ub‐ABPs, the labelling of these enzymes has not been shown in cell lysate.^40^ We tested two methods for lysing cells (Figure 7). The first method used 0.5 % Triton X‐100 (detergent), and with this lysis mode we were able to detect a weak yet convincing level of labelling of endogenous UBE3C in HEK293T (Figure 7 A) and HeLa cells (Figure 7 B). In contrast, we could not detect any labelling for NEDD4, HECTD1, or HUWE1. Importantly, E2–Ub‐ABPs retained specificity, as shown by the absence of labelling with the corresponding F62A mutant probe, which failed to produce the higher molecular‐weight band for UBE3C (indicative of successful labelling). In the second method, we used a sonication protocol without detergent, and obtained more‐robust labelling for endogenous UBE3C. This is in line with other studies that used sonication for cell lysis.^40^, ^42^ Although our assay is non‐quantitative, addition of 2 mm DTT during labelling seemed to slightly increase UBE3C labelling in both HEK293T and HeLa cells (Figure 7 C). As in Figure 7 A, His‐UBE2D2_F62A‐ABP did not result in any labelling, thus emphasising the specificity of the probe. In contrast to UBE3C, which could be readily labelled without any cell stimulation, labelling could not be detected for NEDD4, HECTD1, HUWE1, or UBR5.

Having validated a protocol for labelling endogenous UBE3C in cell lysate with His‐UBE2D2‐ABP, we then evaluated the labelling of Ub‐ABPs with HeLa cell lysate (Figure 7 D). In addition to His‐UBE2D2‐ABP, which showed strong labelling, we also observed a band that (given its size) would correspond to endogenous UBE3C modified with Ub‐PA, but not with Ub‐VME. This is in line with the in vitro assays, which also showed that Ub‐PA might be better at labelling GST‐UBE3C (Figure 5 C). Although labelling of endogenous HECTD1 could not be detected, we wondered whether overexpression of the full‐length enzyme might improve the detection of any labelled pool of the enzyme. HA‐FL‐mouse‐HECTD1 was transiently overexpressed in HEK293T cells. Following sonication, the cell lysate was incubated with each ABP (5 μm, Figure 7 E). UBE3C was used as positive control. However, we could not detect any labelling of HA‐FL‐mHECTD1, so this suggests that (under basal conditions at least) the active‐site Cys is not accessible for efficient labelling. This might imply that HECTD1 and the other HECT ligases have low intrinsic activity in cells under basal/unstimulated conditions.

## Discussion and Conclusion

The development of novel chemical biology tools over the last two decades has contributed to our understanding of the cellular functions and the biochemical/structural properties of DUBs. Ubiquitin‐aldehyde, the first Ub‐based ABP, was initially used as a general inhibitor of Cys‐based DUBs.^23^ More recently, new‐generation Ub‐ABPs have been developed with different C‐terminal electrophilic warheads. In addition, N‐terminal modifications with biochemical (e.g., HA, His) or cellular (e.g., Cy5 fluorophore) tags have enabled these chemical tools to be used in a variety of assays aimed at deciphering the function of DUBs.^27^, ^41^, ^52^ Ub‐VME has been used for DUB profiling in order to identify novel putative deubiquitylating enzymes in cells. The development of novel warheads, in particular Ub‐PA, has improved the specificity of ABPs towards DUBs.^26^ Structural studies of DUBs have revealed multiple Ub‐binding interfaces on the catalytic domain to account for DUB‐linkage specificity, and these efforts have led to the generation of new di‐ubiquitin‐based ABPs, which also offer increased specificity for the targeted DUB.^53^ The latest Ub‐based ABP, Ub‐Dha, undergoes *trans*‐thioesterification, and cascades from E1 to E2 and finally E3 without being transferred to the substrate. Therefore, this mechanism‐based ligase probe offers a unique opportunity to monitor the activities of components of the ubiquitylation cascade upon drug treatment and for certain pathological cues and stresses.^42^

In order to assess with more specificity the activity of HECT and RBR ubiquitin ligases, a new type of ABP based on E2 rather than ubiquitin was recently developed.^40^ These E2–Ub‐ABPs are based on an activated vinylsulfide electrophile (AVS), which is included between E2 and Ub and forms the basis for the activity‐based labelling of catalytic Cys nucleophiles in E3 ligases. Although these probes have great potential to provide novel insights on RBR E3 ubiquitin ligases through functional cellular assays and structural studies, these probes have not yet been extensively explored for their use with HECT E3 ubiquitin ligases. Our data show that the HECT domains of NEDD4, UBE3C, and HECTD1 can be labelled in vitro by E2–Ub‐ABPs. Given that specific E2 ligases preferentially support the activity of particular HECT ligases, these E2‐based ABPs could be used to target HECT ligases with more specificity.^40^, ^41^ For example, our data show that UBE2D2 is more efficient at supporting HECTD1 ligase activity than UBE2L3. In vitro assays confirmed previous findings that the HECT domain of HUWE1 can be labelled by Ub‐VME^39^ and Ub‐PA.^26^ We now extend these observations to show that Ub‐VME efficiently labels the HECT domains of NEDD4, UBE3C, HECTD1, and UBR5; Ub‐PA labels NEDD4, UBE3C, and HUWE1. The use of ABPs is summarised in Table 1. The time‐course labelling experiment shows that E2–Ub‐ABPs quickly and efficiently label the HECT domain of NEDD4, thus suggesting that these reagents are particularly powerful to capture transient E3 ligase activity under specific cellular conditions.

**Table 1:** Summary of the compatibility between ABPs (including recently developed Ub‐Dha)^42^ and HECT ubiquitin ligases.

HECT ubiquitin ligase	Activity‐based probes	Ref.
NEDD4	His‐UBE2L3–Ub (**7**)	this study
	His‐UBE2L3–Ub (**8**)	this study
	His‐UBE2D2–Ub	this study
	Ub‐VME	this study
	biotin‐Ub‐PA	this study
	Ub‐Dha	42
UBE3C	His‐UBE2L3–Ub (**7**)	this study
	His‐UBE2L3–Ub (**8**)	this study
	His‐UBE2D2–Ub	this study (also with cell lysate)
	Ub‐VME	this study
	biotin‐Ub‐PA	this study (also with cell lysate)
	Ub‐Dha	42
HUWE1	Ub‐VME	39 (also with cell lysate); this study
	biotin‐Ub‐PA	26; this study
HECTD1	His‐UBE2D2–Ub	this study
	Ub‐VME	this study
	Ub‐Dha	42 (also with cell lysate)
UBR5	Ub‐VME	this study

UBE3C is found at the proteasome where it functions as an E4 to extend short Ub chains on difficult‐to‐degrade substrates.^51^, ^54^, ^55^ The fact that His‐UBE2D2‐ABP can efficiently label UBE3C, but not proteasomal DUBs yUbp6, UCH37 or yRpn8–11, suggests that these E2–Ub‐ABPs might act as specific tools to inhibit and/or detect the activity of this proteasome‐resident E3 ubiquitin ligase. Furthermore, we have validated the use of ABPs in cell lysate. Specifically, we showed that endogenous UBE3C can be labelled with E2–Ub‐ABPs (and to a lesser extent Ub‐PA) under basal/unstimulated culture conditions in HEK293T and HeLa cells. Therefore, both ABPs could potentially be used as tools to dissect further the fate and function of Ub chains extended by this E4 at the proteasome.^51^ In contrast to UBE3C, we did not detect labelling of endogenous NEDD4, HUWE1, HECTD1, or UBR5 in HEK293T or HeLa cells under basal/unstimulated condition. This could be attributable the limit of sensitivity of western blotting, especially if only a small pool of enzyme has been modified.

However, the absence of labelling more likely reflects the low activity of some HECT ligases under basal/unstimulated conditions. In support of this, for the first time, E2–Ub‐ABPs were successfully used to demonstrate activation of the endogenous RBR E3 ubiquitin ligase Parkin in response to mitochondrial depolarization.^40^ Furthermore, NEDD4 ligase activation was shown to require release of the C2 domain, which can be achieved through NEDD4‐interacting proteins NDFIP1 and NDFIP2 or by calcium.^56^, ^57^ More recently, HUWE1 ligase activity was shown to be regulated by conformational changes and that p14ARF could regulate HUWE1 ligase activity by maintaining it in an autoinhibited state.^58^ The activity of Itch was shown to be activated through a phosphorylation‐induced conformational change,^59^ and the activity of WWP2 was regulated by the polymerizing state of Dishevelled, a key component of Wnt signalling.^60^ Therefore, a better understanding of how the activity of individual HECT ligases is regulated at the molecular level, together with the development of novel ABPs, will be instrumental in dissecting the cellular roles and functions of these enzymes in different cellular contexts. Furthermore, although the crystal structures of some members of the HECT family have been solved (E6AP,^10^ WWP1,^61^ SMURF2,^43^ NEDD4‐L,^38^ HUWE1,^62^ yeast Rsp5,^36^ NEDD4,^37^ and WWP2),^63^ those of N‐end‐rule pathway (UBR5) and the ubiquitin fusion degradation (UFD) pathway (TRIP12, HECTD1) lack structural knowledge. ABPs could accelerate this, and in doing so they could increase our understanding of how specific Ub chains are assembled. The recent emergence of E2–Ub‐ABPs and the novel Ub‐ABP Ub‐Dha greatly expand the Ub toolbox and provide new ways to decipher the cellular functions and structural/biochemical properties of HECT ligases.

Our work provides important new insights for monitoring HECT ligase activity in specific cellular contexts as well as potentially in normal and disease states.^40^ For example, UBE3C appears to be overexpressed in glioma tissue, and this is thought to promote glioma progression through inhibition of the tumour suppressor gene ANXA7.^64^ ABPs might be particularly useful here to monitor UBE3C ligase activity in the search for better therapeutics and to further dissect its mechanism of action during cancer progression.

## Experimental Section

**Sequence alignment**: Sequences for the human HECT domains of E6AP, HUWE1, NEDD4, UBE3C, HECTD1 and UBR5 were retrieved from UniProt and aligned with T‐COFFEE.^65^ Secondary structure was predicted for E6AP by using PHD secondary structure prediction methods (https://npsa‐prabi.ibcp.fr/cgi‐bin/npsa_automat.pl?page=/NPSA/npsa_phd.html) and further analysed onto the ESPript server.^66^ Sequences for human UBE2L3 (UBCH7), UBE2D1 (UBCH5A), UBE2D2 (UBCH5B), and UBE2D3 (UBCH5C) were retrieved and aligned as above.

**Plasmids**: GST‐tagged HECT domains from NEDD4 (AA521–900) cloned from the African green monkey cell lines COS‐7 (HECT domain of NEDD4 is fully conserved in human and corresponds to AA940‐1319), human UBE3C (AA664–1083), human HUWE1 (AA3993–4374) have been previously described,^67^ and human UBR5 (AA2217‐2799) was a kind gift from Dr. Nikola Novcic (MRC‐LMB, Cambridge). Human HECTD1 (AA2129‐end) was amplified (forward: 5′‐CCAAT TGGAT CCAAG CATGA AAGAG TAAAA GTTCC ACGTG G‐3′; reverse 5′‐CCTTG GCTCG AGTCA ATTGA GATGA AAGCC TTTCT CCATT GTAG‐3′) by using cDNA from Normal Human Bronchial Epithelial cell line (NHBE) and cloned into pGEX‐6P‐1 with BamH1 (5′) and Xho1 (3′). pBG100 encoding His‐UBCH7 (UBE2L3) was a gift from Dr. Stefan Bagby (University of Bath), primer sequences and constructs for pGEX‐6P‐1 vectors encoding UBE2D1 (UBCH5A), UBE2D2 (UBCH5B), and UBE2D3 (UBCH5C) are available on request.

**Recombinant proteins**: The TRABID AnkOTU domain was expressed and purified as previously described.^48^ His‐UBE1 (E‐304), OTUD3_CD (E‐574), and UCH37 (E‐327) were obtained from R&D Systems (Minneapolis, MN). Purified recombinant Yeast Ubp6 and Rpn8‐11 were kindly provided by Professor Michael Glickman (Technion‐Israel Institute of Technology).^49^

**Protein expression and purification**: GST‐tagged HECT domains, GST‐tagged UBE2D1, ‐2, and ‐3 and His‐UBE2L3 were expressed in *E. coli* and purified by affinity chromatography. GST‐HECT domains and His‐UBE2L3 were eluted with reduced glutathione (10 mm) or imidazole (500 mm), respectively, prior to desalting on a HiTrap Desalting 1×5 mL column or gel filtration. The GST tag was removed from GST‐UBE2Ds by incubation with PreScission protease. Fractions were concentrated (1 mg mL^−1^) and quantified with a NanoDrop 2000c spectrophotometer (Thermo Fisher Scientific), and stocks were aliquoted, flash‐frozen in liquid nitrogen, and stored at −80 °C.

**In Vitro auto‐ubiquitylation assays**: The mixture consisted of His‐UBE1 (100 ng), E2 (500 ng), GST‐HECT E3 ubiquitin ligase (2.5 μg), and bovine ubiquitin (2.5 μg, U6253, Sigma–Aldrich) in Tris (10 μL, 25 mm, pH 7.4) with NaCl (20 mm), MgCl_2_ (10 mm), DTT (1 mm), and ATP (1 mm). Reactions were carried out at 30 °C for 3 h and stopped by addition of 4×LDS Sample Buffer with DTT (100 mm). Samples were analysed by immunoblotting.

In order to ensure that modified UBE2D2 (which forms part of the E2–ubiquitin ABP) is functional in supporting HECT ligase activity of GST‐HECTD1, GST‐UBR5, and GST‐HUWE1, ubiquitylation assays were also carried out with either His‐UBE2D2_3xCys_WT (C21S, C107S, C11S, catalytic Cys) or His‐UBE2D2_3xCys_F62A (C21S, C107S, C11S, catalytic Cys; F62A). These mutated E2s were kindly provided by Dr. Satpal Virdee (MRC‐PPU, University of Dundee).

**Immunoblotting**: Samples were resolved on 4–12 % SDS‐PAGE under reducing conditions and transferred to a polyvinylidene difluoride membrane (PVDF, 0.45 μm, Thermo Fisher Scientific) or Immobilon FL (Merck Millipore) for near‐infrared fluorescence detection by an Odyssey Clx (LI‐COR, Bad Homburg, Germany). Membranes were blocked in non‐fat dried skimmed milk powder (5 %, *w*/*v*) in PBST with Tween‐20 (0.1 %) for 1 h at RT. Membranes were then probed with the appropriate primary antibodies in blocking buffer overnight at 4 °C. Detection was performed by incubating membranes with HRP‐conjugated or IRDye secondary antibodies in blocking buffer at RT for 1 h. Enhanced chemiluminescence (ECL; Thermo Fisher Scientific) was used for anti‐ubiquitin western blots, and images were acquired on a FUSION‐SL imager (Vilber Lourmat, Marne‐la‐Vallée, France). Anti‐GST blots were visualized on a LI‐COR Clx.

**Antibodies**: Primary antibodies were anti‐ubiquitin rabbit polyclonal antibody (#07‐375, EMD Millipore), anti‐GST goat polyclonal antibody (#27‐4577‐01, GE Healthcare), anti‐His HRP‐conjugated antibody (#A7058, Sigma–Aldrich), and anti‐HA antibody (clone 3F10, Roche). The following antibodies were used to detect endogenous HECT ligases: ab101992 (Abcam, Cambridge, UK; for HECTD1), ab14592 (for NEDD4), ab101512 (for UBE3C; but A304‐122A (Bethyl Laboratories, Montgomery, TX) used for Figure 7 D), ab70161 (for HUWE1), and 8H10D10 (anti‐actin; Cell Signaling Technology, Danvers, MA). PIERCE high‐sensitivity streptavidin‐HRP (Thermo Fisher Scientific) was used to detect biotin‐Ub‐PA (also referred to as Ub‐PA in the main text). HRP‐conjugated secondary antibodies used for ECL‐based detection were Sc‐2054 (goat anti‐rabbit IgG), Sc‐2005 (goat anti‐mouse IgG), Sc‐2032 (goat anti‐rat IgG), all from Santa Cruz Biotechnology (Santa Cruz, CA). IRDye 680RD Donkey anti‐goat secondary antibody (#925‐68074; LI‐COR) was used for Clx detection.

**Ubiquitin‐ABPs**: Ub‐VME and biotin‐Ub‐PA were kindly contributed by Prof. Huib Ovaa (Leiden University). of GST‐HECT or DUB (2 μm) was incubated with Ub‐VME or biotin‐Ub‐PA (10 μm) for 8 h at 30 °C. Reactions were quenched by addition of 4×LDS/DTT sample buffer. Samples were resolved by 4–12 % SDS‐PAGE and detected by Coomassie staining (Coomassie Brilliant Blue R‐250 in methanol (50 %) and acetic acid (10 %), followed by de‐stained with a methanol (40 %)/acetic acid (10 %), or by silver staining (ProteoSilver, Sigma–Aldrich).

**E2–ubiquitin‐ABPs**: His‐UBE2DL3–Ub‐ABP (WT and F63A), His‐UBE2D2–Ub‐ABP (WT and F62A) were kindly provided by Dr. Satpal Virdee. GST‐HECT E3 ubiquitin ligase or DUB (2 μm) was incubated with E2–Ub‐ABPs (10 μm) for 8 h (unless otherwise stated) at 30 °C.^40^ Probe labelling was detected by Coomassie or silver staining. Immunoblotting used HRP‐conjugated anti‐His antibody to detect GST‐HECTs labelled with E2–Ub‐ABPs. In order to ensure that the labelling of the GST‐HECT was specific, E2–Ub‐ABP mutants carrying a single point mutation were used (F63A for His‐UBE2L3‐ABP, probes **7 F** and **8 F**; F62A for His‐UBE2D2‐ABP).^40^

**Labelling of endogenous HECTs in cell lysate**

**Lysis with Triton X‐100**: HEK293T and HeLa cells were grown in DMEM supplemented with FBS (10 %) and penicillin/streptomycin (100 U mL^−1^) at 37 °C in 5 % CO_2_. Cells from three 10 cm plates for each cell line were harvested at subconfluence in PBS. Cells were then lysed on ice for 15 min in labelling buffer (Tris (200 μL, 50 mm, pH 7.4) with sucrose (0.27 mm), EDTA (5 μm), and a cOmplete protease inhibitor tablet) with Triton X‐100 (0.5 %). Lysates were then cleared by centrifugation in an Eppendorf 5417R microcentrifuge (13 000 rpm, 15 min, 4 °C). Supernatant (25 μL) was used in a reaction mixture (30 μL) containing activity‐based probe (5 μm) (or no ABP). Following incubation for 3 h at 30 °C, labelling reactions were stopped by addition of 4×LDS/DTT (30 μL). Samples were then resolved by 4–12 % PAGE and detected by western blotting.

**Sonication**: Cells were grown and harvested as described above. Cell pellets were resuspended in ice‐cold labelling buffer and subjected to sonication in a Branson Sonifier (15 cycles of 0.3 s on 1 s off, amplitude 55 %). Lysates were then clarified by centrifugation as described above (13 000 rpm, 15 min, at 4 °C). Clarified lysate (25 μL) was used in a reaction mixture (30 μL) containing activity‐based probe (5 μm) (or no ABP), in the presence or absence of DTT (2 mm). Following incubation for 3 h at 30 °C, labelling reactions were stopped by addition of 4×LDS/DTT(30 μL). Samples were then run on 4–12 % SDS‐PAGE and detected by western blotting.

**Labelling of overexpressed HECTD1 in cell lysate**: HEK293T cells were grown in 10 cm dishes and transfected by using Lipofectamine 2000, at 80 % confluency, with HA‐Full‐Length‐mouse‐HECTD1 (5 μg). After 48 h, cells were pelleted, washed in PBS, and lysed by sonication. Labelling and analysis of samples were as above.

## Conflict of interest

*The authors declare no conflict of interest*.
